# Clinical and Pathologic Features of H-Type Bovine Spongiform Encephalopathy Associated with E211K Prion Protein Polymorphism

**DOI:** 10.1371/journal.pone.0038678

**Published:** 2012-06-08

**Authors:** Justin J. Greenlee, Jodi D. Smith, M. Heather West Greenlee, Eric M. Nicholson

**Affiliations:** 1 National Animal Disease Center, United States Department of Agriculture, Agricultural Research Service, Ames, Iowa, United States of America; 2 Iowa State University, Ames, Iowa, United States of America; The Scripps Research Institute Scripps Florida, United States of America

## Abstract

The majority of bovine spongiform encephalopathy (BSE) cases have been ascribed to the classical form of the disease. H-type and L-type BSE cases have atypical molecular profiles compared to classical BSE and are thought to arise spontaneously. However, one case of H-type BSE was associated with a heritable E211K mutation in the prion protein gene. The purpose of this study was to describe transmission of this unique isolate of H-type BSE when inoculated into a calf of the same genotype by the intracranial route. Electroretinograms were used to demonstrate preclinical deficits in retinal function, and optical coherence tomography was used to demonstrate an antemortem decrease in retinal thickness. The calf rapidly progressed to clinical disease (9.4 months) and was necropsied. Widespread distribution of abnormal prion protein was demonstrated within neural tissues by western blot and immunohistochemistry. While this isolate is categorized as BSE-H due to a higher molecular mass of the unglycosylated PrP^Sc^ isoform, a strong labeling of all 3 PrP^Sc^ bands with monoclonal antibodies 6H4 and P4, and a second unglycosylated band at approximately 14 kDa when developed with antibodies that bind in the C-terminal region, it is unique from other described cases of BSE-H because of an additional band 23 kDa demonstrated on western blots of the cerebellum. This work demonstrates that this isolate is transmissible, has a BSE-H phenotype when transmitted to cattle with the K211 polymorphism, and has molecular features that distinguish it from other cases of BSE-H described in the literature.

## Introduction

Prion diseases or transmissible spongiform encephalopathies (TSEs) are fatal neurodegenerative diseases that naturally affect several species including human beings. These chronic diseases are associated with the accumulation of a protease-resistant, disease-associated isoform of the prion protein (PrP^Sc^) in the central nervous system and other tissues, depending on the species affected. In humans, TSEs can be acquired through exposure to infectious material, inherited as germline polymorphisms in the prion gene (*prnp*), or occur spontaneously.

Bovine spongiform encephalopathy (BSE), a TSE of cattle, can be subdivided into at least three groups: classical, H-type, and L-type. The latter 2 designations are based on higher or lower apparent molecular mass profiles of the unglycosylated PrP^Sc^ band in a western blot [1], [2], [3] and are collectively referred to as atypical BSE. The vast majority of BSE cases are the classical subtype that was associated with the UK BSE epizootic. Classical BSE has spread to several mammalian species including human beings [4], [5], [6] causing major animal health and public health concerns.

Epidemiological studies suggest that classical BSE is spread through contaminated feedstuffs, and early in the UK epizootic it was suspected that the origin of the disease was scrapie [7], [8], a TSE known to exist in sheep for over 200 years. However, experimental transmission of scrapie to cattle by a natural route has failed to produce disease, and while transmission of scrapie to cattle by the intracranial route produces a disease in cattle, it fails to accurately reproduce the clinical and pathologic features of BSE in cattle [9], [10]. Thus the origin of classical BSE remains unclear.

H-type BSE has been described in cattle from France [11], Germany [1], Japan [12], the Netherlands [13], Poland [14], Switzerland [15], the United Kingdom [16], Canada [17], and the United States [18]. The molecular phenotype of the H-type BSE cases is characterized by 1) a higher molecular mass of the unglycosylated PrP^Sc^ isoform, 2) a strong labeling of all 3 PrP^Sc^ polypeptides (unglycosylated, monoglycosylated and diglycosylated isoforms) with the PrP-specific monoclonal antibodies 6H4 and P4, and 3) a second unglycosylated band at approximately 14 kDa when developed with antibodies that bind in the C-terminal region (amino acids 154–236) such as SAF 84 [19].

Several hypotheses have been proposed to explain atypical BSE cases [11]. At the forefront of this discussion is the possibility that both H-type and L-type BSE may be spontaneous diseases in cattle. Support for atypical BSE occurring spontaneously is drawn from the parallels to sporadic TSE in humans, specifically, occurrence in older hosts and a comparable low incidence rate. Whereas numbers of cattle affected by classical BSE shows a striking response to control measures, numbers of cattle affected by atypical BSE remain relatively stable over time supporting the hypothesis that these atypical cases occur spontaneously [20]. Furthermore, cases of atypical BSE are not influenced by prion gene promoter region polymorphisms that influence susceptibility to classical BSE [21], and atypical BSE occurs as isolated cases in contrast to the clustering of cases observed for feedborne classical BSE [22]. Recognition of a spontaneous TSE in cattle, coupled with evidence indicating atypical BSE can convert to classical BSE upon serial passage in mice [23], [24], [25], has broad implications for our understanding of the origins of the classical form of the disease.

In 2006, a case of BSE was diagnosed in the U.S. that led to the identification of a new prion protein (*prnp*) allele, K211 [26], that is associated with H-type BSE and is heritable [27]. There was limited historical information available for this case and only brainstem tissue was available for analysis. The purpose of this study was to describe the transmission of a unique isolate of H-type BSE derived from the 2006 U.S. case associated with the E211K prion protein polymorphism when inoculated into a calf of the same genotype. Clinical signs, antemortem test results, histopathology, and tissue distribution of PrP^Sc^ by western blot (WB) and immunohistochemistry (IHC) are reported here.

## Results and Discussion

### Clinical Findings

A calf with the K211 allele was intracranially inoculated with H-type BSE from the US 2006 BSE case that also had one K211 allele. This calf demonstrated clinical signs at approximately 9.4 months (288 days) post-inoculation (PI). Initial signs were non-descript: listlessness, head down in non-physiologic position with drooping ears, and decreased feed consumption. Within a week, clinical signs had progressed to the calf separating himself from others in the pen, head pressing into the wall or gate, and intermittent reluctance to rise with a stumbling gate for a brief time after rising. The calf began to demonstrate a lip licking and accentuated chewing behavior that was not associated with feeding. The lip-licking and chewing behaviors increased in frequency and severity, and at the time of necropsy at approximately 9.8 months PI (301 days), the calf was depressed, salivating excessively, and reluctant to rise.

Progression to severe clinical signs of BSE occurred in this animal after 9.8 months, a faster onset than the 12–18 months described for other experimental cases of H-type BSE [28], [29], [30], [31]. Previous studies describe the onset of clinical signs for BSE-H as early as 8 months PI [31], but more commonly at 12 months PI [30] or later [28] with a 2–7 month progression of disease to ataxia and inability to rise [28], [30], [31]. Our findings are similar to other reports in that the earliest clinical signs appear to be vague: weight loss, depression, and low head carriage. However, reports of clinical findings in BSE-H are variable: from ataxia and myoclonus that progresses to an inability to rise without nervousness or aggression [30] to a nervous disease form that is characterized by overeactivity to external stimuli, apprehension and anxiety [31]. This case of E211K BSE-H is different in that the most obvious outward clinical signs were bizarre licking and chewing behaviors not described elsewhere. While the calf affected with E211K was reluctant to rise, it was able to rise when encouraged, however, this animal was younger and smaller than cattle in other studies that had difficulty getting to their feet, which may play a role in the difference reported.

### Electroretinography

**Figure 1:**
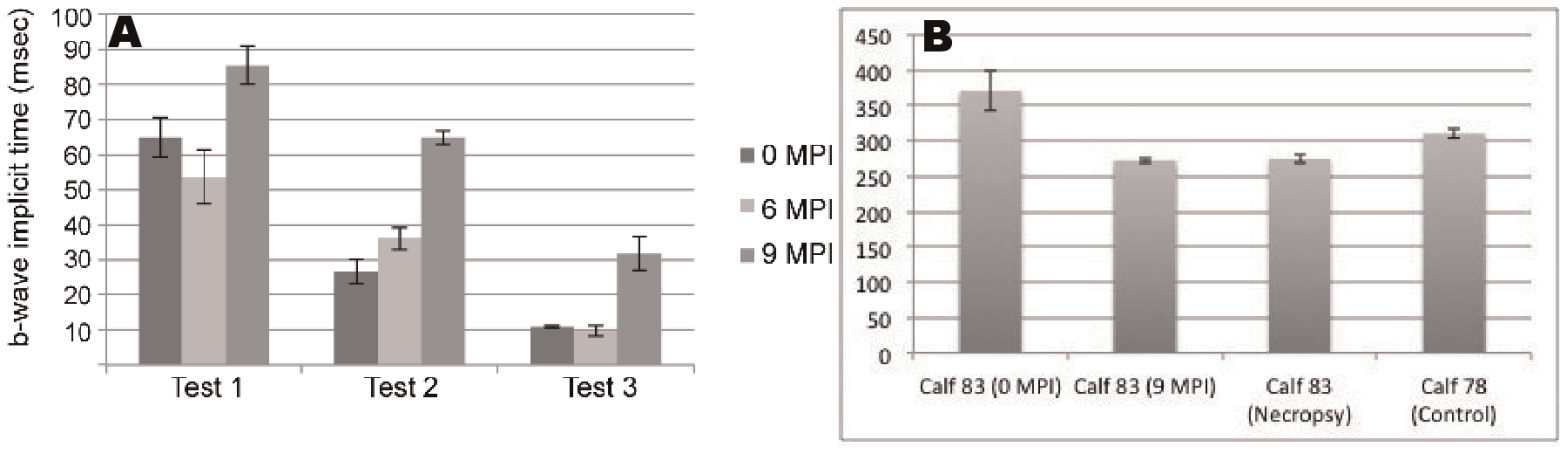
Results of antemortem ophthalmologic testing. (**A**)Comparison of mean ERG b-wave implicit time at 0, 6, and 9 months post-inoculation (MPI). Implicit time was prolonged at 9 MPI compared to baseline (0 MPI) and 6 MPI values for all test conditions. Test 1, dark adapted 0.024 cd•s/m^2^; test 2, dark adapted 2.45 cd•s/m^2^; test 3, light adapted 2.45 cd•s/m^2^. (**B**) Optical Coherence Tomography (OCT) was used to measure full retinal thickness, (including all retinal layers) of Calf 83 prior to inoculation (0 MPI), nine-months post inoculation (9 MPI) and one day prior to necropsy (approximately 9.8 MPI). There was an appreciable decrease in retinal thickness from 0 MPI to 9 MPI. Retinal thickness was also decreased relative to an age-matched, genotype matched, non-inoculated control (Calf 78). Error bars are standard deviation based on 10 measurements. Abbreviations: cd•s/m^2^ = candela seconds per meter squared; msec = milliseconds.

As the retina has proven to offer diagnostic promise in TSEs [32], [33], two non-invasive tools were utilized to assess retinal changes during the incubation period. Retinal function and thickness were assessed through electroretinography and optical coherence tomography (OCT), respectively. Flash electroretinography was performed prior to inoculation (0 months post-inoculation; MPI)) and at 6 and 9 MPI. Data at the 9 MPI time-point was collected approximately 1 week prior to the onset of unequivocal neurologic signs. B-wave amplitude and implicit time were measured for 2 scotopic (dark-adapted) tests (tests 1,2) and 1 photopic (ambient light) test (test 3) (Fig. 1). B-wave amplitude increased for all tests from 0 to 6 MPI, but did not appreciably change from 6 to 9 MPI, which included the time of onset of clinical signs (Table S1). However, the average b-wave implicit time increased substantially over the course of disease. For tests 1, 2, and 3 at 0 MPI, average b-wave implicit times were 65 msec (±6), 27 msec (±4), and 10.8 msec (±0.3), respectively. By 9 MPI, implicit time for each test was substantially prolonged with average values for tests 1, 2, and 3 at 86 msec (±5), 65 msec (±2.0), and 31.8 msec (±5), respectively (Fig. 1). These findings are consistent with our previous report demonstrating that while b-wave amplitude is not affected, b-wave implicit time is prolonged in cattle with prion disease [33].

### Optical Coherence Tomography and Retinal Pathology

Retinal thickness was measured using optical coherence tomography (OCT) 1 day prior to inoculation (0 MPI), at 9 MPI and immediately prior to necropsy. At 0 MPI, the average retinal thickness measurement was 371 µm (±27). At 9 MPI the retinal thickness had decreased to an average of 273 µm (±4). One month later, immediately prior to necropsy, average retinal thickness was not appreciably different at 275 µm (±6) (Fig. 1B). To ensure that decreased retinal thickness could be attributed to disease and not to the normal retinal thinning that accompanies growth of the globe, we compared the retinal thickness measurements of the calf with an age-matched non-inoculated control calf that also had the E211K polymorphism. The average retinal thickness of this control animal was 310 µm (±6), suggesting that an appreciable amount of the retinal thinning observed in the inoculated calf was associated with the disease process. While this is the first published report using OCT in cattle, the retinal thinning observed is consistent with prior reports of morphologic change associated with disease [32], [33], [34], [35], [36], [37].

**Figure 2:**
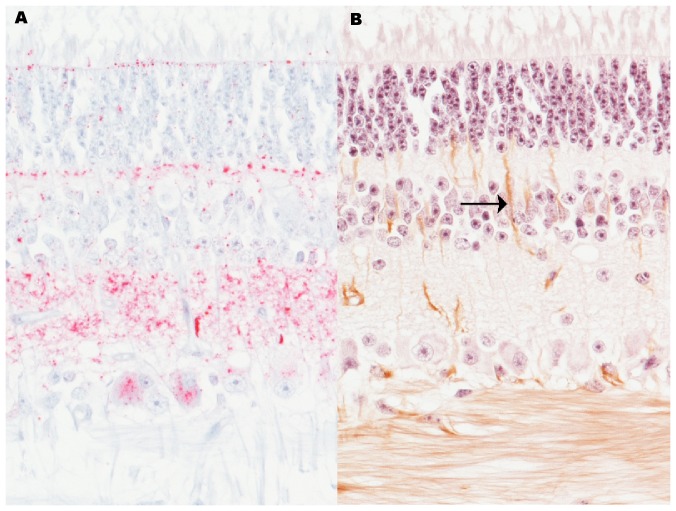
Retinal pathology corroborates antemortem findings. (**A**) PrP^Sc^ immunoreactivity demonstrates that abundant PrP^Sc^ (red) accumulates in the retina. Original magnification 20×. (**B**) GFAP immunoreactivity (brown) in the Müller glia (arrow) indicates a response to retinal injury. Original magnification 20×.

Immunohistochemical analysis of retina demonstrated evidence of PrP^Sc^ accumulation and a retinal response to injury. PrP^Sc^ immunoreactivity in the retina was intense within the plexiform layers and in the retinal ganglion cells (Fig. 2A). In addition to PrP^Sc^, the left and right globes were also labeled for glial fibrillary acidic protein (GFAP), a cytoskeletal protein that is upregulated in activated Müller glia. GFAP immunoreactivity was used to further assess the retinal injury related to disease. Compared to a non-inoculated bovine control, GFAP-immunoreactivity was increased in the retina of this calf (Fig. 2B). These findings are consistent with reports of scrapie in sheep [34] and TME in cattle [33].

### Distribution and Characterization of Lesions in BSE-H

**Figure 3:**
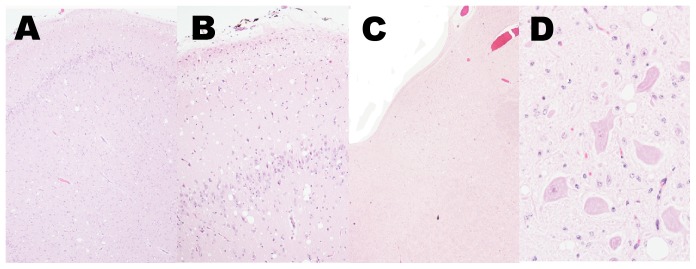
Spongiform change was present at all levels of the brain examined. (**A, B**) Vacuoles were numerous and evenly spread in the piriform cortex. Original magnification 4× and 10×, respectively. (**C, D**) The parasympathetic nucleus of the vagus nerve contained few but definitive spongiform lesions. Vacuoles occur predominantly in the neuropil rather than perikarya. Original magnification 2× and 40×, respectively.

Vacuolar lesions typical of spongiform encephalopathy were present throughout the brain of this calf. Spongiform change was most severe in the piriform cortex and hippocampus, but present at all levels of brain examined. The distribution of lesions suggests sampling at various levels of the brain, including the obex, would be fruitful for diagnosis. Vacuolation scores ranged from 1 to 3 (scale of 0 to 4), but the vast majority of regions were scored a 2 or higher, indicating definitive spongiform lesions (Fig. S1). Lesions predominantly affected gray matter with little to no involvement of white matter. Vacuoles were primarily present in the neuropil, but were also detected within the cytoplasm of neurons (Fig. 3). At all levels of the spinal cord, there were few inconclusive vacuoles present in the neuropil of the dorsal horns.

Results of microscopic examination for vacuolar change indicate that additional tools may be required to differentiate E211K BSE from classical BSE or other isolates of BSE-H that have been described in the literature [30], [31]. Similar to other reports, vacuolar change was generally observed in all brain areas and moderate to severe vacuolar change was detected in cerebral cortex, cerebellum, basal ganglia, thalamus, and brainstem [30], [31]. However, there were contrasts in the areas with the highest vacuolation scores. The highest levels of spongiform change were evident in piriform cortex and hippocampus in this case, whereas the highest levels were in thalamic nuclei and midbrain of other reports [30]. Profiles developed using larger numbers of animals suggest that BSE-H may be difficult to distinguish from classical BSE based on spongiform change in the obex, but may have increased numbers of vacuoles in rostral brain areas [31]. E211K BSE-H had the lowest scores in pontine and hypoglossal motor nuclei, which was similar to previous reports of BSE-H [30]. In summary, it appears that vacuolar change is variable amongst different isolates classified as BSE-H. Caution should be used when considering the lesion profile of this single animal as what role individual animal differences or the E211K polymorphism play cannot be determined without further experimentation.

Microscopic evaluation of the brain of the US 2006 H-type BSE case was limited to the obex and complicated by freeze artifact, precluding a definitive microscopic interpretation [18]. Therefore, this is the first description of the microscopic lesions in the CNS of a bovid affected with H-type BSE associated with the E211K polymorphism. No amyloid plaques were present in the tissues from the calf with E211K BSE-H, which is similar to one previous study of BSE-H [31], but contrasts with another [30].

**Figure 4:**
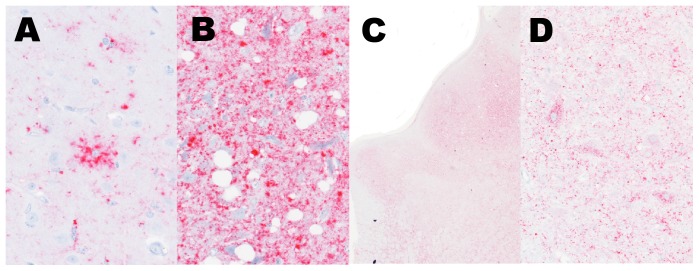
Patterns of immunoreactivity (red) in the brain vary depending on region. Staining patterns observed included (**A**) intraglial, stellate in the cerebrum, (**B**) coalescing throughout the neuropil of the hippocampus, and (**C, D**) intraneuronal in the perikarya of hypoglossal nucleus. Original magnification 40×, 40×, 2×, and 20×, respectively.

Immunohistochemical analysis for PrP^Sc^ demonstrated widespread immunoreactivity throughout the brain, spinal cord, and retina with lesser immunoreactivity in neurohypophysis and the trigeminal ganglia (Fig. S2). Regardless of the brain region examined, PrP^Sc^ immunoreactivity was readily apparent. Immunoreactivity was most intense in the brainstem and midbrain and patterns of immunoreactivity were similar to those previously described [1], [18], [26], [30], [31]with an intraglial distribution predominating. The predominant patterns in the cortex were intraglial and stellate on a background of fine punctate and granular particulate staining that was multifocally coalescing (Fig. 4A). Perineuronal staining was also evident, but intraneuronal immunoreactivity was rare. Immunoreactivity increased in intensity from frontal cortex caudal to occipital cortex. In the white matter subjacent to the cortex, there were rare coarse particulate foci of immunoreactivity that were most often associated with glial cell margins (Fig. S3). This is in contrast to previous studies where glial staining in the white matter was a more prominent feature in BSE-H [31]. We did not see PrP^Sc^ immunoreactive plaques in the gray or white matter, but other reports indicate that this occurs as a prominent [30] or lesser [31] feature. Immunoreactivity in hippocampus, midbrain, and brainstem was markedly intense and frequently formed coalescing aggregates (Fig. 4B). While intraneuronal straining was rare in the cortex, it became the most obvious pattern in the midbrain and brainstem nuclei (Fig. 4D) with notable exception of the parasympathetic nucleus of the vagus nerve. Intraneuronal staining also was readily apparent in spinal cord (Fig. S2). Immunoreactivity was scant in the cerebellum where small, multifocal clumps of granular and particulate staining occurred in the molecular and granular layers. The cerebellar white matter was devoid of immunoreactivity except for in association with deep cerebellar nuclei (Fig. S4), which is in contrast to other studies of BSE-H where the most prominent staining of the cerebellum was in the white matter [31]. Considering the strong immunoreactivity in other regions of the brain, the scant immunoreactivity in cerebellum was surprising. This finding corroborates recent studies examining PrP^Sc^ immunoreactivity in the brainstem and cerebellum of cases of BSE-H where the cerebellum and caudal brainstem contained less PrP^Sc^ than more rostral regions of the brainstem [31], [38]The immunohistochemical techniques used here failed to demonstrate PrP^Sc^ in other tissues examined. This finding is consisent with other studies of atypical BSE that suggest that no significant PrPSc depositions occur in peripheral tissues [39]. Other findings of the lesions described contrast those described for wild-type cattle with BSE-H [30] in that no PrP^Sc^ plaques were noted and that there is less immunoreactivity in the cerebellum in this case. Whether the E211K polymorphism influences lesion character or distribution when inoculated with other BSE isolates will require further study.

### Molecular Characterization of E211K BSE

**Figure 5:**
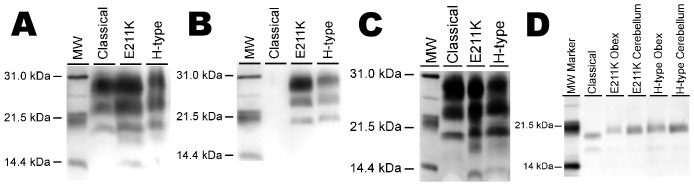
Western blot analysis with a panel of monoclonal antibodies. (**A**) Samples from five brain regions of calf #83 demonstrate the characteristic 3-band profile of PrP^Sc^ when developed with mAb 6H4. The unglycosylated, monoglycosylated, and diglycosylated bands are of similar intensity. Note the presence of an additional band at approximately 23 kDa in the sample from cerebellum. (**B**) Western blot analysis with monoclonal antibody P4 showing the characteristic 3 band profile of PrP^Sc^ for both E211K and BSE-H, but an absence of detectable PrP^Sc^ for classical BSE. (**C**) Western blot analysis with mAb SAF-84 showing comparison of PrP^Sc^ profiles in brain of classical, E211K, and H-type BSE. E211K and H-type BSE have a 4^th^ band at 14 kDa, whereas classical BSE does not. (**D**) All BSE-H samples appear similar after PNGase F treatment (mAb 6H4). Samples were loaded at 0.6–0.8 mg (A–C) or 0.1–0.3 mg (D) equivalents of brain tissue per lane. Molecular weight standards flank the blot and the molecular weight in kDa is indicated to the left of the blot.

Western blot analysis of this E211K BSE-affected calf was performed using monoclonal antibodies 6H4, P4, and SAF-84 alongside control tissues from cattle affected with classical BSE and BSE-H. On western blots labeled with mAb 6H4, cerebrum, thalamus, colliculus, and obex demonstrated the characteristic three-band pattern of prion disease with similar intensity of unglycosylated, monoglycosylated, and diglycosylated bands. Not previously described for BSE-H was the presence of an additional band at approximately 23 kDa in tissue from the cerebellum of calf #83 (Fig. 5A), which is not present on western blots of brain homogenates from the same regions of cattle inoculated with classical BSE or BSE-H. This additional band is also present when mAb P4 is used, but not when primary antibody is omitted (data not shown). Upon deglycosylation, the 23 kDa band is not observed giving the blot an appearance similar to other BSE-H isolates. While mAb P4 does not label PrP^Sc^ from animals with classical BSE, labeling with mAb P4 is a characteristic of BSE-H (Fig. 5B). The use of mAb SAF-84 further demonstrates the BSE-H properties of E211K BSE with the characteristic low molecular weight band of BSE-H at approximately 14 kDa. The 14-kDa band is similarly present in non-E211K BSE-H, but not in classical BSE (Fig. 5C). E211K BSE-H presents all the features necessary for classification as BSE-H, specifically a higher molecular weight banding profile than classical BSE and labeling with mAb P4. However, E211K BSE-H remains distinct from other described BSE-H by both the E211K polymorphism and the presence of an additional (4^th^) band at approximately 23 kDa in samples from the cerebellum when labeled with either mAb P4 or 6H4. The origin of this additional band is not clear, but it may be specific to either the host E211K genotype or to E211K BSE-H inoculum. The 23 kDa band is not present after deglycosylation leaving a single band that appears similar to other BSE-H isolates (Fig. 5D). Passage of E211K BSE-H in normal cattle and passage of non-E211K BSE-H in E211K cattle will both be required to fully address this matter. These findings support the idea that BSE-H represents a spectrum of BSE-isolates that can be categorized by western blot similarities, but are not a single disease.

The disease reported here was true to the molecular characterization of the case diagnosed in 2006, which is the best approximation of H-type BSE that may occur later in life in cattle with the E211K polymorphism. Based on the case history of the original 2006 E211K BSE case and the fact that the vast majority of naturally-occurring atypical BSE cases involve older cattle (>10 yrs of age), we speculate that a pre-clinical period of at least 10 years will be required for BSE-H to naturally occur in E211K cattle without prior exposure to infectious material. While an inoculation study cannot definitely prove that the U.S. 2006 BSE-H case was due to the E211K polymorphism, i.e. an inherited TSE, the results of this study do suggest that cattle with the K211 allele are predisposed to rapid onset of BSE-H when exposed.

Most significantly it must be determined if the molecular phenotype of this cattle TSE remains stable when transmitted to cattle without the E211K polymorphism as several other isolates of atypical BSE have been shown to adopt a molecular profile consistent with classical BSE after passage in transgenic mice expressing bovine PrP^C^ [40] or multiple passages in wild type mice [23]. Results of ongoing studies, namely passage of the E211K H-type isolate into wild-type cattle, will lend further insight into what role, if any, genetic and sporadic forms of BSE may have played in the origins of classical BSE. Atypical cases presumably of spontaneous or, in the case of E211K BSE-H, genetic origins highlight that it may not be possible to eradicate BSE entirely and that it would be hazardous to remove disease control measures such as prohibiting the feeding of meat and bone meal to ruminants.

## Materials and Methods

### Animals and Procedures

This experiment was carried out in accordance with the Guide for the Care and Use of Laboratory Animals (Institute of Laboratory Animal Resources, National Academy of Sciences, Washington, DC) and the Guide for the Care and Use of Agricultural Animals in Research and Teaching (Federation of Animal Science Societies, Champaign, IL). The protocol was approved by the Institutional Animal Care and Use Committee at the National Animal Disease Center (protocol number: 3985). A calf with the E211K polymorphism confirmed by *prnp* sequencing was generated by embryo transfer from the only known female offspring of the US 2006 atypical BSE case [27]. At approximately 2-months-old, it was inoculated intracranially as described previously [41] with 1 ml of 10% (w/v) brain homogenate derived from the 2006 U.S. H-type BSE case associated with the E211K prion protein polymorphism. Briefly, the calf was sedated with xylazine, the frontal area was clipped and scrubbed, a 1 cm midline incision was made in the skin slightly caudal to the junction of the parietal and frontal bones, and a 1 mm hole was drilled through the calvarium. A 22 gauge spinal needle was advanced through the hole perpendicular to the frontal bones until the tip of the needle made contact with the opposite (bottom) side of the calvarium. The inoculum was slowly injected as the needle was withdrawn through the brain. The skin was closed with tissue glue (Vetbond, 3M, St. Paul, MN, USA).

The calf was observed daily for clinical signs of disease. It was euthanized and necropsied when unequivocal signs of TSE were noted (see results). Two sets of tissue samples including representative sections of liver, kidney, spleen, skin, striated muscles (heart, tongue, diaphragm, masseter), thoracic aorta, thyroid gland, turbinates, trachea, lung, tonsils, esophagus, rumen, reticulum, omasum, abomasum, intestines (ileum), adrenal gland, urinary bladder, lymph nodes (retropharyngeal, prescapular, mesenteric, popliteal), tonsils (palatine and nasopharyngeal), nerves (sciatic, optic, trigeminal), pituitary gland, trigeminal ganglion, brain (cerebral cortex, cerebellum, midbrain including superior colliculus, brainstem including obex), spinal cord (cervical, thoracic, lumbar), and eye were collected. The first set was collected into 10% buffered formalin (globes were fixed in Bouin's fixative), embedded in paraffin wax, and sectioned at 5 µm for staining with hematoxylin and eosin (HE) and anti-prion protein antibodies. The second set of tissues was frozen.

### Electroretinography

Electroretinography was performed prior to inoculation and at 6 and 9 months post-inoculation as previously described [33] with slight modification of the testing protocol. An EPIC 4000 visual electrodiagnostic testing system (LKC Technologies, Gaithersburg, MD) with a CMGS-1 Color Mini-Ganzfeld Stimulator (LKC Technologies, Gaithersburg, MD) was used to capture electroretinograms (ERG). The left eye was tested at each time point. The calf was dark adapted for 20 minutes, followed by a series of 3 scotopic recordings (single white flash 0.024 cd•s/m^2^, single white flash 2.45 cd•s/m^2^, oscillatory potentials), 10 minutes of light adaptation, and 2 photopic recordings (single white flash 2.45 cd•s/m^2^ and 59 Hz flicker). B-wave amplitude and implicit time were measured for each single-flash ERG.

### Optical Coherence Tomography

Retinal thickness was measured *in vivo* using optical coherence tomography (OCT). A Bioptigen SD-OCT (Bioptigen, Durham, NC USA) was used to capture linear B scans (10 mm; 1000 A scans/B scan). At each time point at least 10 measurements of retinal thickness were taken from multiple scan frames (using on-screen calipers) to determine an average thickness measurement. The values reported are +/− standard deviation of the measurements.

### Histopathology and Immunohistochemistry

Vacuolation profiles were generated by examining defined regions of brain on hematoxylin and eosin stained sections and scoring the level of vacuolation. Gray matter regions evaluated included the piriform cortex, rostral cerebral cortex, caudate nucleus, thalamic nuclei, rostral (superior) colliculus, hippocampus, caudal (inferior) colliculus, central/periaqueductal gray matter, reticular formation at the level of the pons, pontine nuclei, nodulus and flocculus of the cerebellum, and the parasympathetic nucleus of the vagus nerve, hypoglossal motor nucleus, and reticular formation at the level of the obex. White matter was evaluated in the rostral cerebrum and cerebellum. For gray matter, scores were assigned as follows: 0 = no vacuolation; 1 = occasional vacuole indicative of aging or spongiform encephalopathy (SE), but diagnosis inconclusive; 2 = definitive SE defined as the presence of crisp, round to oval vacuoles within neurons and/or neuropil, but no even spread of vacuoles; 3 = even spread of vacuoles throughout the region with coalescence of some vacuoles; 4 = heavy, even spread of vacuoles throughout the region with frequent coalescence (most severe). For white matter: 0 = no definite SE or occasional inconclusive axonal vacuolation, 1 = definite SE, but no even spread; 2 = even spread of SE throughout the region; 3 = heavy, even spread of SE throughout the region with frequent coalescence (most severe).

All paraffin-embedded tissues were also stained by an automated immunohistochemical method for detection of PrP^Sc^ as described previously [42] with slight modifications. Briefly, after deparaffinization and rehydration, tissue sections were autoclaved for 30 minutes in an antigen retrieval solution (DAKO Target Retrieval Solution, DAKO Corp., Carpinteria, CA) and stained with an indirect, biotin-free staining system containing an alkaline phosphatase labeled secondary antibody (*ultra*view Universal Alkaline Phosphatase Red Detection Kit, Ventana Medical Systems, Inc., Tucson, AZ) designed for an automated immunostainer (NexES IHC module, Ventana Medical Systems, Inc., Tucson, AZ). The primary antibody used was F99/97.6.1 [43] at a concentration of 5 µg/ml, and incubation was carried out at 37°C for 32 minutes. Images for the figures were captured using a Nikon DS camera on a Nikon Eclipse 80*i* microscope. Sections of the brain at the levels of frontal cortex, parietal cortex, occipital cortex, rostral colliculus, obex and spinal cord were examined for patterns of immunoreactivity as previously described for scrapie [44].

### Western Blot

Samples were prepared as a 10% (w/v) (1 mg/10 µl) brain homogenate in 1× homogenization buffer (Prionics AG, Switzerland). The samples were digested with proteinase K using a final enzyme concentration of 4 U/ml (80 µg/ml) at 37°C for 1 hour. The digestion was stopped by addition of Pefabloc to a final concentration of 0.1 mg/ml. Samples were diluted 1∶4 in 4× SDS-PAGE sample and analyzed by standard western blotting procedures. The tissue equivalent for loading was empirically derived with the goal of providing a reasonable signal for each case, and in all cases shown here, was loaded with between 0.6 and 0.8 mg equivalents of brain per lane. Western blot detection was conducted using mouse anti-PrP monoclonal antibodies P4 or 6H4 at a 1∶10,000 dilution (0.1 µg/ml), F99/97.6.1 at a 1∶1000 dilution (1 µg/ml), or SAF 84 at 1∶200 dilution (1 µg/ml) as the primary antibody. A biotinylated sheep anti-mouse secondary antibody at 0.05 µg/ml and a streptavidin-HRP conjugate, were used in conjunction with the ECL Plus detection system and visualized on a Kodak Invivo-F imaging system. Primary antibody incubations were conducted with the membrane at either room temperature for 1 hour or 4°C overnight (≥12 hours). Secondary antibody and streptavidin-HRP conjugate incubations were conducted at room temperature for 1 hour.

Deglycosylation of PrP-res was accomplished using PNGase F (New England Biolabs) with slight modifications from the manufacturer's instructions. Briefly, 5–10 µl of 10% brain homogenate was PK digested as above and denatured in sodium dodecyl sulfate (final concentration 3% w/v) at 100°C for 10 minutes, instead of using the denaturing buffer supplied. The PK-digested, denatured homogenates (5–10 µl) were incubated with PNGase F (final concentration 150 U/µl) at 37°C for ≥16 hours. The samples were combined with loading buffer and heated to 100°C for 5 minutes prior to gel electrophoresis.

## Supporting Information

Figure S1 **Vacuolation scores in gray matter regions of the brain.** The vast majority of brain regions had definitive spongiform lesions (score >1). Brain regions: 1, piriform cortex; 2, rostral cerebral cortex; 3, caudate nucleus; 4, thalamic nuclei; 5, rostral colliculus; 6, hippocampus; 7, caudal colliculus; 8, central/periaqueductal gray matter; 9, pons – reticular formation; 10, pontine nuclei; 11, cerebellum – nodulus; 12, cerebellum – flocculus; 13, obex – parasympathetic nucleus of the vagus nerve; 14, obex – hypoglossal motor nucleus; 15, obex – reticular formation.
(TIF)

Figure S2 **Patterns of PrP^Sc^ immunoreactivity in neurohypophysis, trigeminal ganglion, and spinal cord.** (**A**) Immunoreactivity is present throughout the neurohypophysis, but not in the adenohypophysis (pars intermedia in upper portion of image). Original magnification 20×. (**B**) Weak intraneuronal immunoreactivity is present in a minority of sensory neuron perikarya in the trigeminal ganglion. Original magnification 40×, (**C**) Immunoreactivity is present throughout the gray matter of the spinal cord. Original magnification is 2×. (**D**) In the ventral horn of the spinal cord, immunoreactivity is throughout the neuropil and in lesser amounts within motor neuron perikarya. Original magnification 20×.
(TIF)

Figure S3 **Patterns of PrP^Sc^ immunoreactivity in white matter and cerebellum.** (**A**) Sparse immunoreactivity in the white matter subjacent to the cerebral cortex is primarily cell associated. Original magnification 40×. (**B**) PrP^Sc^ immunoreactivity is present in low amounts in cerebellar molecular and granular layers. Original magnification 20×. (**C**) PrP^Sc^ immunoreactivity is rare in the cerebellar white matter with exception of areas adjacent the deep cerebellar nuclei. Original magnifcation 40×.
(TIF)

Table S1 **Numerical results of electroretinograms.** B-wave amplitude increased for all tests from 0 to 6 MPI, but did not appreciably change from 6 to 9 MPI. The average b-wave implicit time increased substantially over the course of disease. Test 1, dark adapted 0.024 cd•s/m^2^; test 2, dark adapted 2.45 cd•s/m^2^; test 3, light adapted 2.45 cd•s/m^2^. Abbreviations: A = amplitutde; IT = implicit time; cd•s/m^2^ = candela seconds per meter squared; msec = milliseconds.
(DOCX)

## References

[1] Buschmann A, Gretzschel A, Biacabe AG, Schiebel K, Corona C (2006). Atypical BSE in Germany-proof of transmissibility and biochemical characterization.. Vet Microbiol.

[2] Beringue V, Bencsik A, Le Dur A, Reine F, Lai TL (2006). Isolation from cattle of a prion strain distinct from that causing bovine spongiform encephalopathy.. PLoS Pathog.

[3] Casalone C, Zanusso G, Acutis P, Ferrari S, Capucci L (2004). Identification of a second bovine amyloidotic spongiform encephalopathy: molecular similarities with sporadic Creutzfeldt-Jakob disease.. Proc Natl Acad Sci U S A.

[4] Will RG, Ironside JW, Zeidler M, Cousens SN, Estibeiro K (1996). A new variant of Creutzfeldt-Jakob disease in the UK.. Lancet.

[5] Bruce ME, Will RG, Ironside JW, McConnell I, Drummond D (1997). Transmissions to mice indicate that ‘new variant’ CJD is caused by the BSE agent.. Nature.

[6] Collinge J (1999). Variant Creutzfeldt-Jakob disease.. Lancet.

[7] Wilesmith JW, Wells GA, Cranwell MP, Ryan JB (1988). Bovine spongiform encephalopathy: epidemiological studies.. Vet Rec.

[8] Wilesmith JW, Ryan JB, Atkinson MJ (1991). Bovine spongiform encephalopathy: epidemiological studies on the origin.. Vet Rec.

[9] Cutlip RC, Miller JM, Race RE, Jenny AL, Katz JB (1994). Intracerebral transmission of scrapie to cattle.. J Infect Dis.

[10] Cutlip RC, Miller JM, Hamir AN, Peters J, Robinson MM (2001). Resistance of cattle to scrapie by the oral route.. Can J Vet Res.

[11] Biacabe AG, Laplanche JL, Ryder S, Baron T (2004). Distinct molecular phenotypes in bovine prion diseases.. EMBO Rep.

[12] Sugiura K, Onodera T, Bradley R (2009). Epidemiological features of the bovine spongiform encephalopathy epidemic in Japan.. Rev Sci Tech.

[13] Biacabe A-G, Jacobs JG, Bencsik A, Langeveld JPM, Baron TGM (2007). H-Type Bovine Spongiform Encephalopathy.. Prion.

[14] Jacobs JG, Langeveld JP, Biacabe AG, Acutis PL, Polak MP (2007). Molecular discrimination of atypical bovine spongiform encephalopathy strains from a geographical region spanning a wide area in Europe.. J Clin Microbiol.

[15] Tester S, Juillerat V, Doherr MG, Haase B, Polak M (2009). Biochemical typing of pathological prion protein in aging cattle with BSE.. Virol J.

[16] Stack M, Focosi-Snyman R, Cawthraw S, Davis L, Jenkins R (2009). Two unusual bovine spongiform encephalopathy cases detected in Great Britain.. Zoonoses Public Health.

[17] Dudas S, Yang J, Graham C, Czub M, McAllister TA (2010). Molecular, biochemical and genetic characteristics of BSE in Canada.. PLoS ONE.

[18] Richt JA, Kunkle RA, Alt D, Nicholson EM, Hamir AN (2007). Identification and characterization of two bovine spongiform encephalopathy cases diagnosed in the United States.. J Vet Diagn Invest.

[19] Gavier-Widen D, Noremark M, Langeveld JP, Stack M, Biacabe AG (2008). Bovine spongiform encephalopathy in Sweden: an H-type variant.. J Vet Diagn Invest.

[20] Biacabe AG, Morignat E, Vulin J, Calavas D, Baron TG (2008). Atypical bovine spongiform encephalopathies, France, 2001–2007.. Emerg Infect Dis.

[21] Brunelle BW, Hamir AN, Baron T, Biacabe AG, Richt JA (2007). Polymorphisms of the prion gene promoter region that influence classical bovine spongiform encephalopathy susceptibility are not applicable to other transmissible spongiform encephalopathies in cattle.. J Anim Sci.

[22] Donnelly CA, Ghani AC, Ferguson NM, Anderson RM (1997). Recent trends in the BSE epidemic.. Nature.

[23] Capobianco R, Casalone C, Suardi S, Mangieri M, Miccolo C (2007). Conversion of the BASE prion strain into the BSE strain: the origin of BSE?. PLoS Pathog.

[24] Baron T, Vulin J, Biacabe AG, Lakhdar L, Verchere J (2011). Emergence of classical BSE strain properties during serial passages of H-BSE in wild-type mice.. PLoS ONE.

[25] Torres JM, Andreoletti O, Lacroux C, Prieto I, Lorenzo P (2011). Classical bovine spongiform encephalopathy by transmission of h-type prion in homologous prion protein context.. Emerg Infect Dis.

[26] Richt JA, Hall SM (2008). BSE case associated with prion protein gene mutation.. PLoS Pathog.

[27] Nicholson EM, Brunelle BW, Richt JA, Kehrli ME, Greenlee JJ (2008). Identification of a heritable polymorphism in bovine PRNP associated with genetic transmissible spongiform encephalopathy: evidence of heritable BSE.. PLoS ONE.

[28] Balkema-Buschmann A, Ziegler U, McIntyre L, Keller M, Hoffmann C (2011). Experimental challenge of cattle with German atypical bovine spongiform encephalopathy (BSE) isolates.. J Toxicol Environ Health A.

[29] Balkema-Buschmann A, Eiden M, Hoffmann C, Kaatz M, Ziegler U (2011). BSE infectivity in the absence of detectable PrPSc accumulation in the tongue and nasal mucosa of terminally diseased cattle.. J Gen Virol.

[30] Okada H, Iwamaru Y, Imamura M, Masujin K, Matsuura Y (2011). Experimental H-type bovine spongiform encephalopathy characterized by plaques and glial- and stellate-type prion protein deposits.. Vet Res.

[31] Konold T, Bone GE, Clifford D, Chaplin MJ, Cawthraw S (2012). Experimental H-type and L-type bovine spongiform encephalopathy in cattle: observation of two clinical syndromes and diagnostic challenges.. BMC Vet Res.

[32] Smith JD, Greenlee JJ, Hamir AN, West Greenlee MH (2008). Retinal cell types are differentially affected in sheep with scrapie.. J Comp Pathol.

[33] Smith JD, Greenlee JJ, Hamir AN, Richt JA, Greenlee MH (2009). Retinal function and morphology are altered in cattle infected with the prion disease transmissible mink encephalopathy.. Vet Pathol.

[34] Greenlee JJ, Hamir AN, West Greenlee MH (2006). Abnormal prion accumulation associated with retinal pathology in experimentally inoculated scrapie-affected sheep.. Vet Pathol.

[35] Hortells P, Monzon M, Monleon E, Acin C, Vargas A (2006). Pathological findings in retina and visual pathways associated to natural Scrapie in sheep.. Brain Res.

[36] Smith JD, Greenlee JJ, Hamir AN, Greenlee MH (2009). Altered electroretinogram b-wave in a Suffolk sheep experimentally infected with scrapie.. Vet Rec.

[37] Regnier A, Andreoletti O, Albaric O, Gruson DC, Schelcher F (2011). Clinical, electroretinographic and histomorphometric evaluation of the retina in sheep with natural scrapie.. BMC Vet Res.

[38] Polak MP, Zmudzinski JF (2012). Distribution of a pathological form of prion protein in the brainstem and cerebellum in classical and atypical cases of bovine spongiform encephalopathy.. Vet J.

[39] Balkema-Buschmann A, Fast C, Kaatz M, Eiden M, Ziegler U (2011). Pathogenesis of classical and atypical BSE in cattle.. Prev Vet Med.

[40] Torres M, Castillo K, Armisen R, Stutzin A, Soto C (2010). Prion protein misfolding affects calcium homeostasis and sensitizes cells to endoplasmic reticulum stress.. PLoS ONE.

[41] Hamir AN, Kunkle RA, Miller JM, Bartz JC, Richt JA (2006). First and second cattle passage of transmissible mink encephalopathy by intracerebral inoculation.. Vet Pathol.

[42] Hamir AN, Kunkle RA, Richt JA, Miller JM, Cutlip RC (2005). Experimental transmission of sheep scrapie by intracerebral and oral routes to genetically susceptible Suffolk sheep in the United States.. J Vet Diagn Invest.

[43] O'Rourke KI, Baszler TV, Besser TE, Miller JM, Cutlip RC (2000). Preclinical diagnosis of scrapie by immunohistochemistry of third eyelid lymphoid tissue.. J Clin Microbiol.

[44] Gonzalez L, Martin S, Begara-McGorum I, Hunter N, Houston F (2002). Effects of agent strain and host genotype on PrP accumulation in the brain of sheep naturally and experimentally affected with scrapie.. J Comp Pathol.

