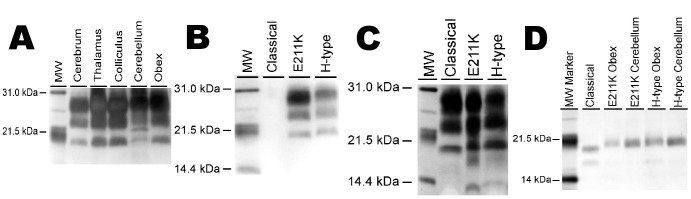# Correction: Clinical and Pathologic Features of H-Type Bovine Spongiform Encephalopathy Associated with E211K Prion Protein Polymorphism

**DOI:** 10.1371/annotation/256348ad-e2eb-40ef-8f0c-fa14475ea5fb

**Published:** 2013-01-17

**Authors:** Justin J. Greenlee, Jodi D. Smith, M. Heather West Greenlee, Eric M. Nicholson

There was an error in Figure 5, panel A. Please see the corrected Figure 5 here: 

**Figure:**