# Supplementary figures and images for: Clinical and Pathologic Features of H-Type Bovine Spongiform Encephalopathy Associated with E211K Prion Protein Polymorphism
Source: PLoS One. 2012 Jun 8;7(6):e38678. doi: 10.1371/journal.pone.0038678 (PMC3371052; doi:10.1371/journal.pone.0038678)

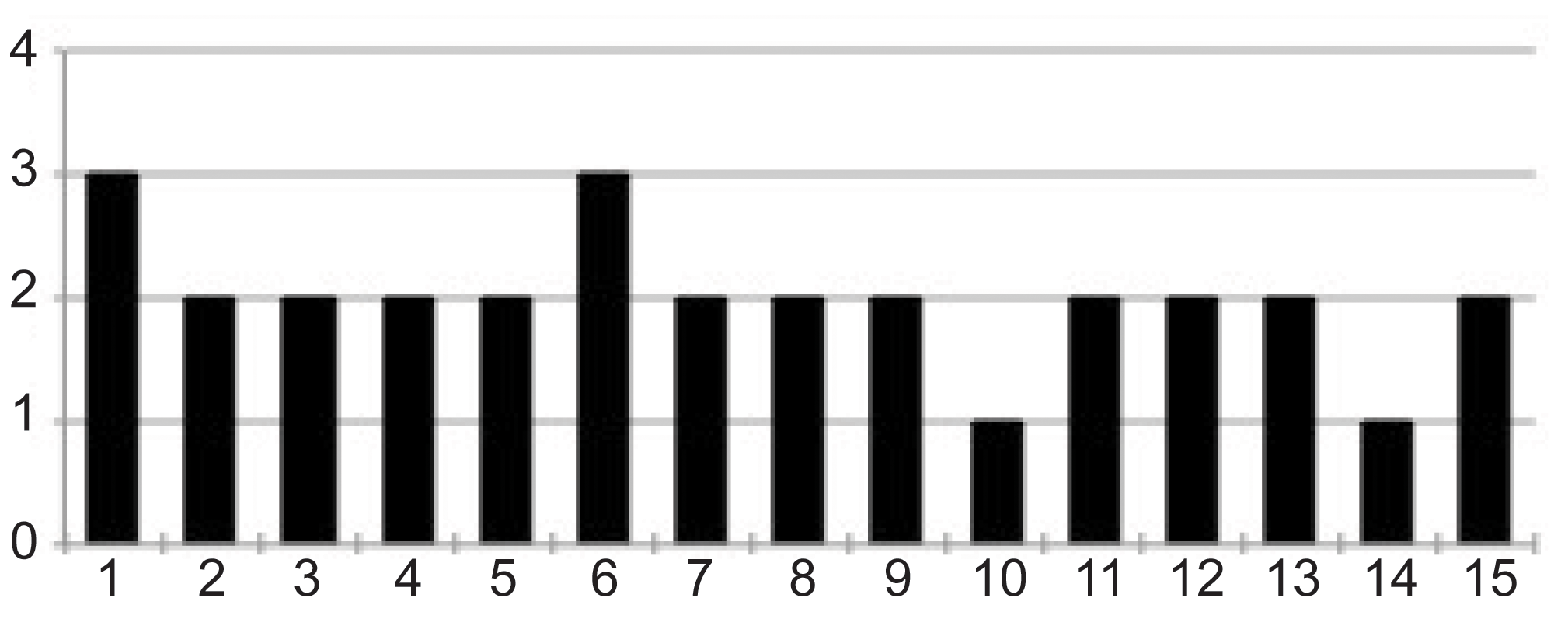

Supplement: Figure S1 — Vacuolation scores in gray matter regions of the brain. The vast majority of brain regions had definitive spongiform lesions (score >1). Brain regions: 1, piriform cortex; 2, rostral cerebral cortex; 3, caudate nucleus; 4, thalamic nuclei; 5, rostral colliculus; 6, hippocampus; 7, caudal colliculus; 8, central/periaqueductal gray matter; 9, pons – reticular formation; 10, pontine nuclei; 11, cerebellum – nodulus; 12, cerebellum – flocculus; 13, obex – parasympathetic nucleus of the vagus nerve; 14, obex – hypoglossal motor nucleus; 15, obex – reticular formation. (TIF) [file pone.0038678.s001.tif]

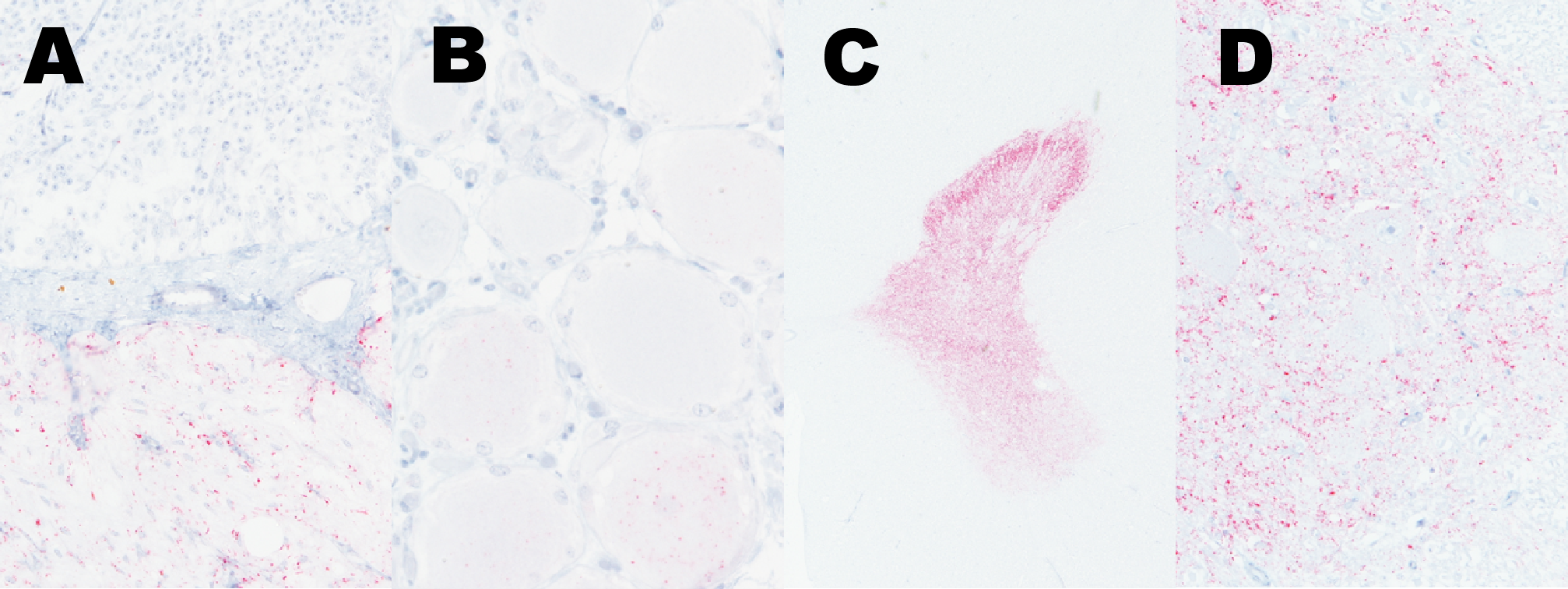

Supplement: Figure S2 — Patterns of PrPSc immunoreactivity in neurohypophysis, trigeminal ganglion, and spinal cord. (A) Immunoreactivity is present throughout the neurohypophysis, but not in the adenohypophysis (pars intermedia in upper portion of image). Original magnification 20×. (B) Weak intraneuronal immunoreactivity is present in a minority of sensory neuron perikarya in the trigeminal ganglion. Original magnification 40×, (C) Immunoreactivity is present throughout the gray matter of the spinal cord. Original magnification is 2×. (D) In the ventral horn of the spinal cord, immunoreactivity is throughout the neuropil and in lesser amounts within motor neuron perikarya. Original magnification 20×. (TIF) [file pone.0038678.s002.tif]

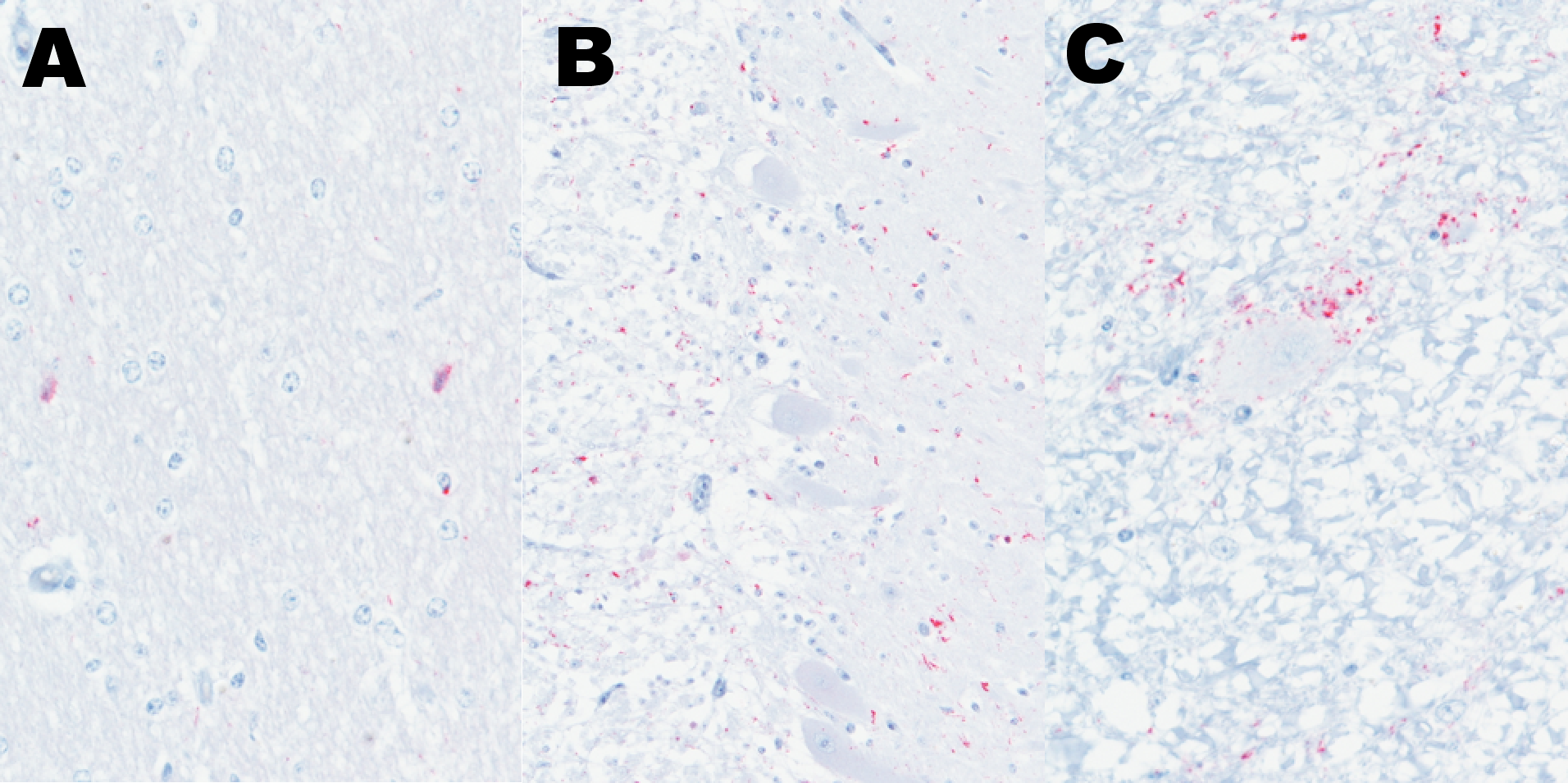

Supplement: Figure S3 — Patterns of PrPSc immunoreactivity in white matter and cerebellum. (A) Sparse immunoreactivity in the white matter subjacent to the cerebral cortex is primarily cell associated. Original magnification 40×. (B) PrPSc immunoreactivity is present in low amounts in cerebellar molecular and granular layers. Original magnification 20×. (C) PrPSc immunoreactivity is rare in the cerebellar white matter with exception of areas adjacent the deep cerebellar nuclei. Original magnifcation 40×. (TIF) [file pone.0038678.s003.tif]
