# Supplementary material for: Clinical and Pathologic Features of H-Type Bovine Spongiform Encephalopathy Associated with E211K Prion Protein Polymorphism
Source: PLoS One. 2012 Jun 8;7(6):e38678. doi: 10.1371/journal.pone.0038678 (PMC3371052; doi:10.1371/journal.pone.0038678)
Supplement: Table S1 — Numerical results of electroretinograms. B-wave amplitude increased for all tests from 0 to 6 MPI, but did not appreciably change from 6 to 9 MPI. The average b-wave implicit time increased substantially over the course of disease. Test 1, dark adapted 0.024 cd•s/m2; test 2, dark adapted 2.45 cd•s/m2; test 3, light adapted 2.45 cd•s/m2. Abbreviations: A = amplitutde; IT = implicit time; cd•s/m2 = candela seconds per meter squared; msec = milliseconds. (DOCX) [file pone.0038678.s004.docx]

**Supplementary Table 1. Numerical results of electroretinograms.**

|  | 0 MPI | | 6 MPI | | 9 MPI | |
| --- | --- | --- | --- | --- | --- | --- |
|  | A | IT | A | IT | A | IT |
| Test 1  (0.024 cd•s/m^2^) | 148 µV ± 44 | 65 msec ± 6 | 218 µV ± 38 | 54 msec ± 8 | 215 µV ± 5 | 86 msec ± 5 |
| Test 2  (2.45 cd•s/m^2^)  Dark Adapted | 223 μV ± 62 | 27 msec ± 4 | 608 µV ± 79 | 36 msec ± 3 | 677 µV ± 14 | 65 msec ± 2.0 |
| Test 3  (2.45 cd•s/m^2^)  Light Adapted | 51 μV ± 5 | 10.8 msec ± 0.3 | 92 µV ± 19 | 9.7 msec ± 1 | 124 µV ± 16 | 31.8 msec ± 5 |

B-wave amplitude increased for all tests from 0 to 6 MPI, but did not appreciably change from 6 to 9 MPI. The average b-wave implicit time increased substantially over the course of disease. Test 1, dark adapted 0.024 cd•s/m^2^; test 2, dark adapted 2.45 cd•s/m^2^; test 3, light adapted 2.45 cd•s/m^2^.

Abbreviations: A=amplitutde; IT=implicit time; cd•s/m^2^ = candela seconds per meter squared; msec= milliseconds.
